# Supplementary figures and images for: Metabolic regulation of misfolded protein import into mitochondria
Source: eLife. 2024 Jun 20;12:RP87518. doi: 10.7554/eLife.87518 (PMC11189628; doi:10.7554/eLife.87518)

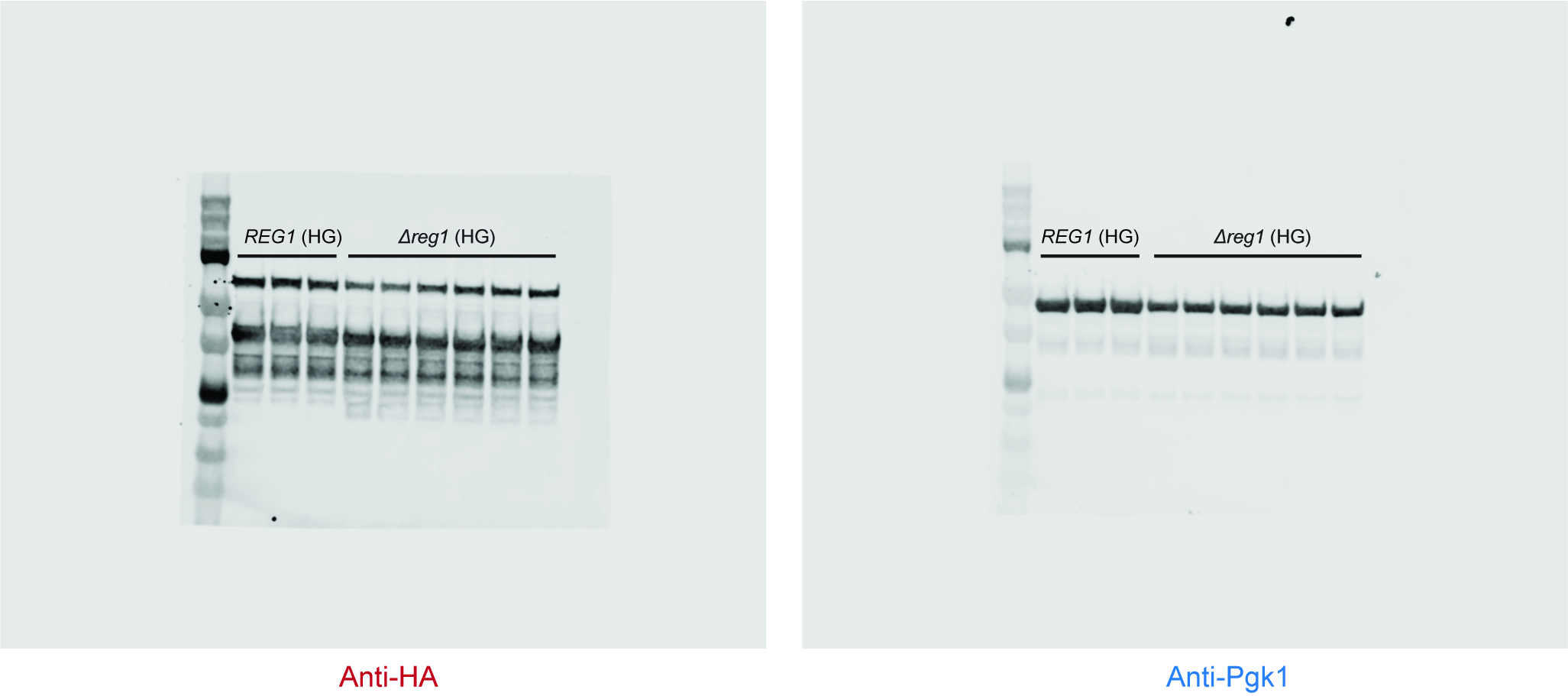

Supplement: Figure 1—figure supplement 2—source data 2. [file elife-87518-fig1-figsupp2-data2.zip › Figure 1-Figure Supplement 2-Source Data 2/REG1 vs reg1 null in HG media/Labeled blots.tif]

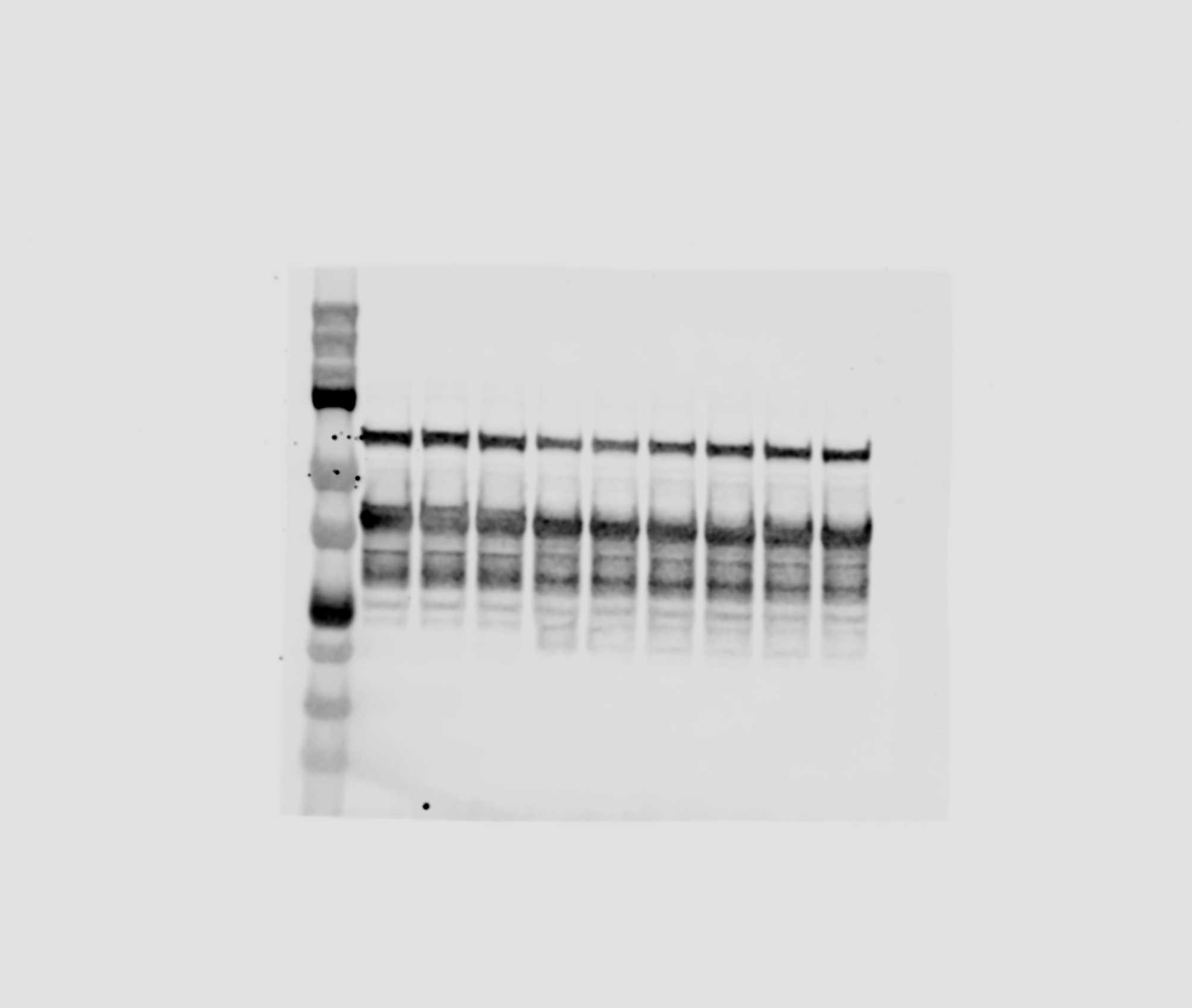

Supplement: Figure 1—figure supplement 2—source data 2. [file elife-87518-fig1-figsupp2-data2.zip › Figure 1-Figure Supplement 2-Source Data 2/REG1 vs reg1 null in HG media/REG1 HG vs reg1 HG_anti-HA.tif]

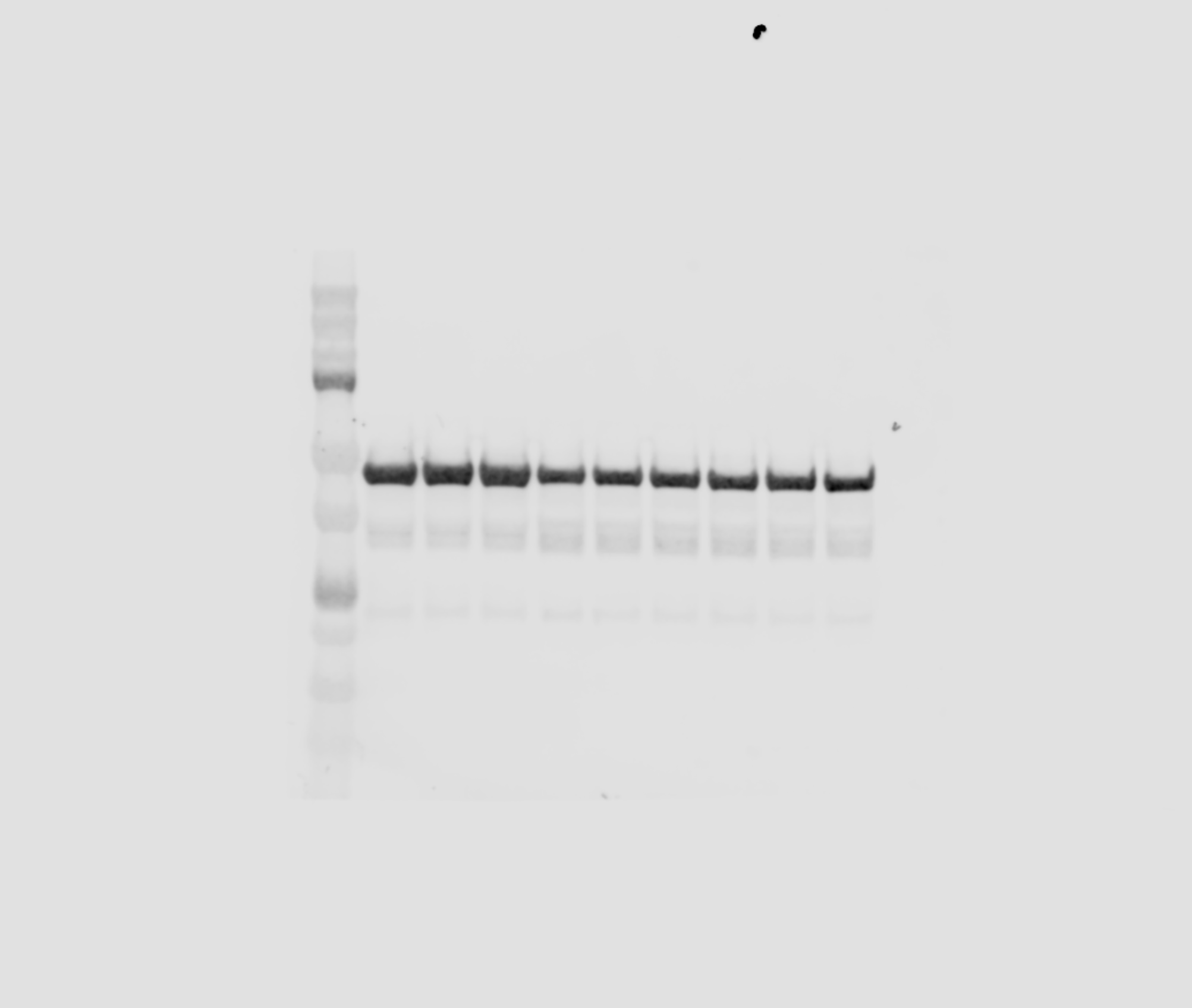

Supplement: Figure 1—figure supplement 2—source data 2. [file elife-87518-fig1-figsupp2-data2.zip › Figure 1-Figure Supplement 2-Source Data 2/REG1 vs reg1 null in HG media/REG1 HG vs reg1 HG_anti-Pgk1.tif]

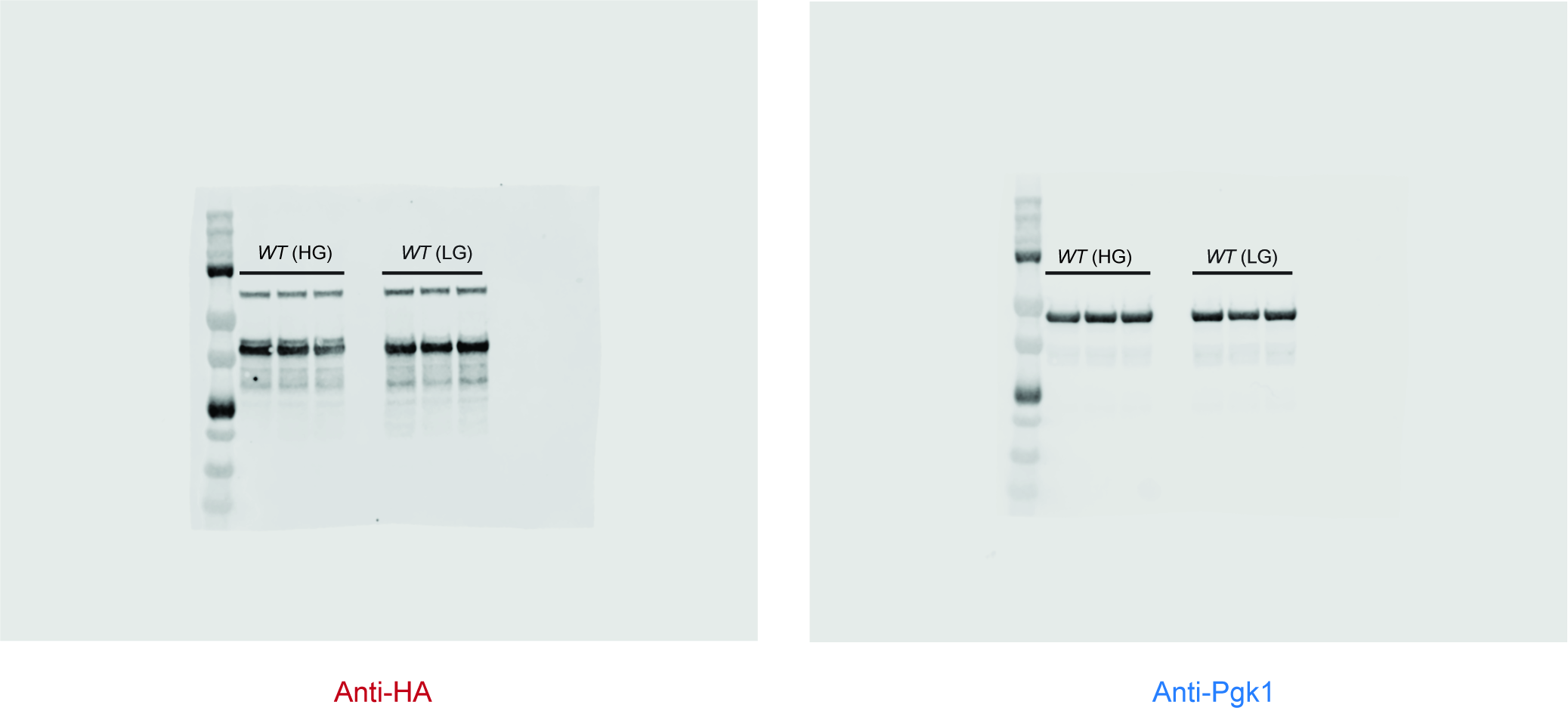

Supplement: Figure 1—figure supplement 2—source data 2. [file elife-87518-fig1-figsupp2-data2.zip › Figure 1-Figure Supplement 2-Source Data 2/WT in HG vs LG media/Labeled blots.tif]

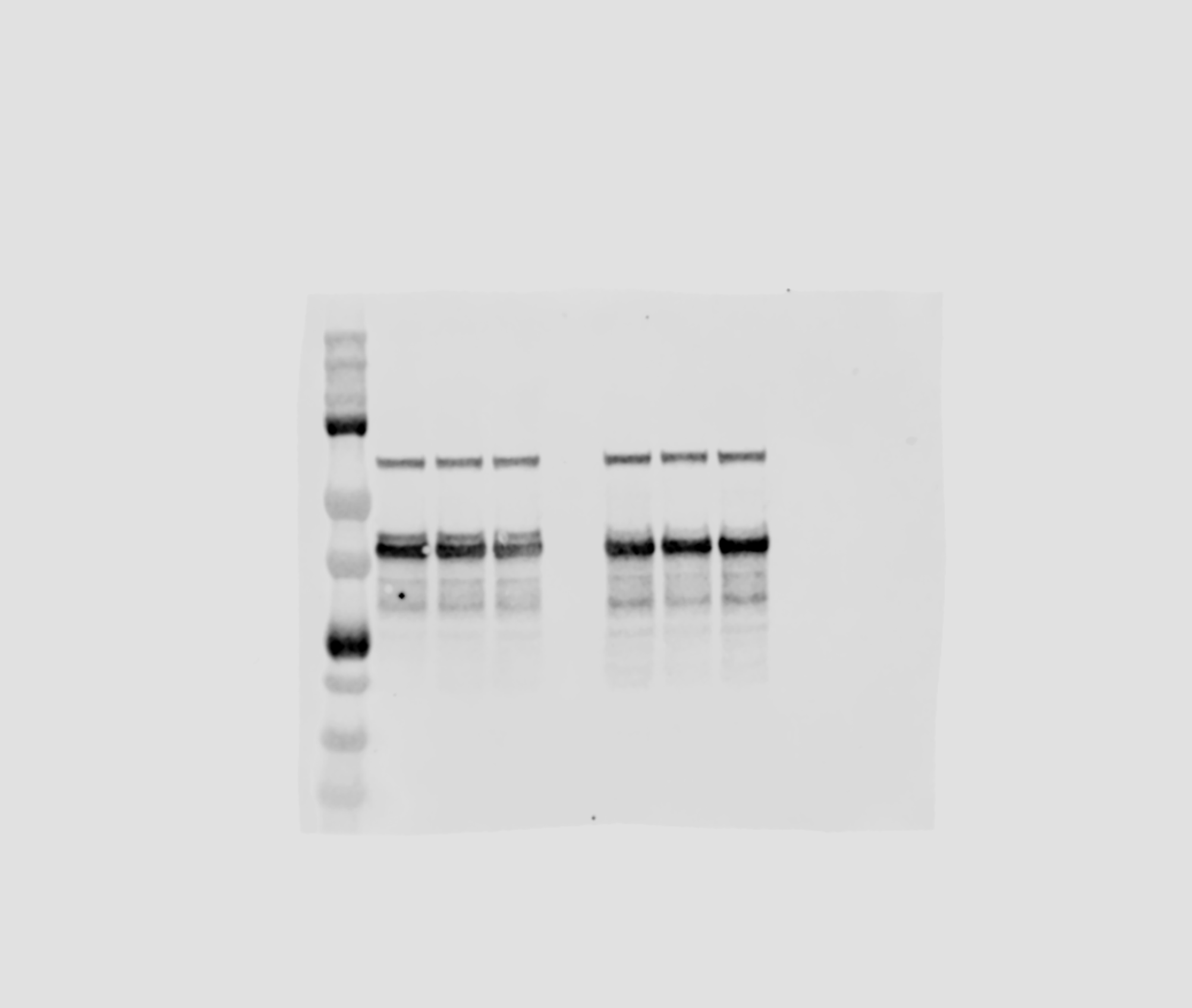

Supplement: Figure 1—figure supplement 2—source data 2. [file elife-87518-fig1-figsupp2-data2.zip › Figure 1-Figure Supplement 2-Source Data 2/WT in HG vs LG media/WT HG vs WT LG_anti-HA.tif]

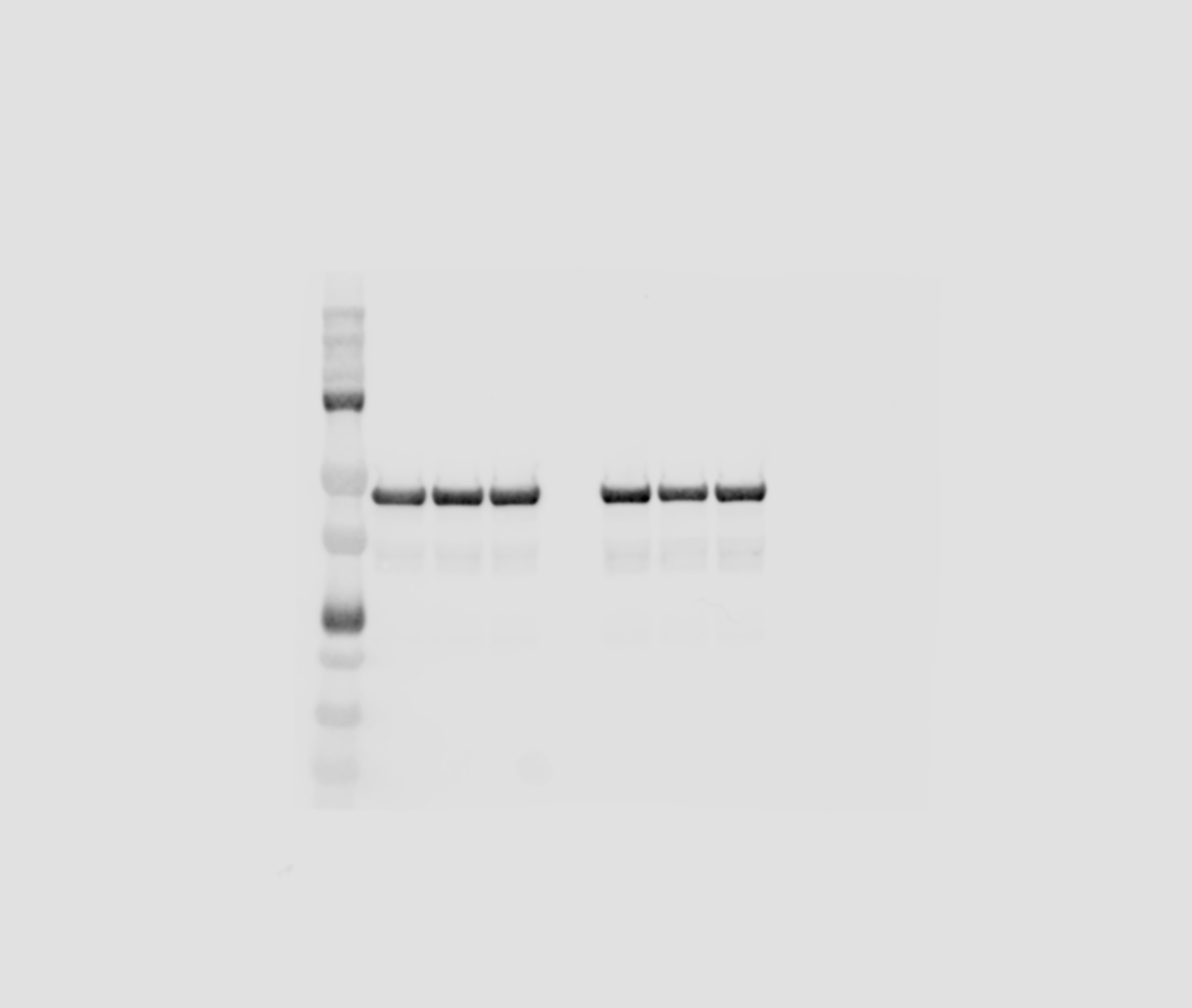

Supplement: Figure 1—figure supplement 2—source data 2. [file elife-87518-fig1-figsupp2-data2.zip › Figure 1-Figure Supplement 2-Source Data 2/WT in HG vs LG media/WT HG vs WT LG_anti-Pgk1.tif]

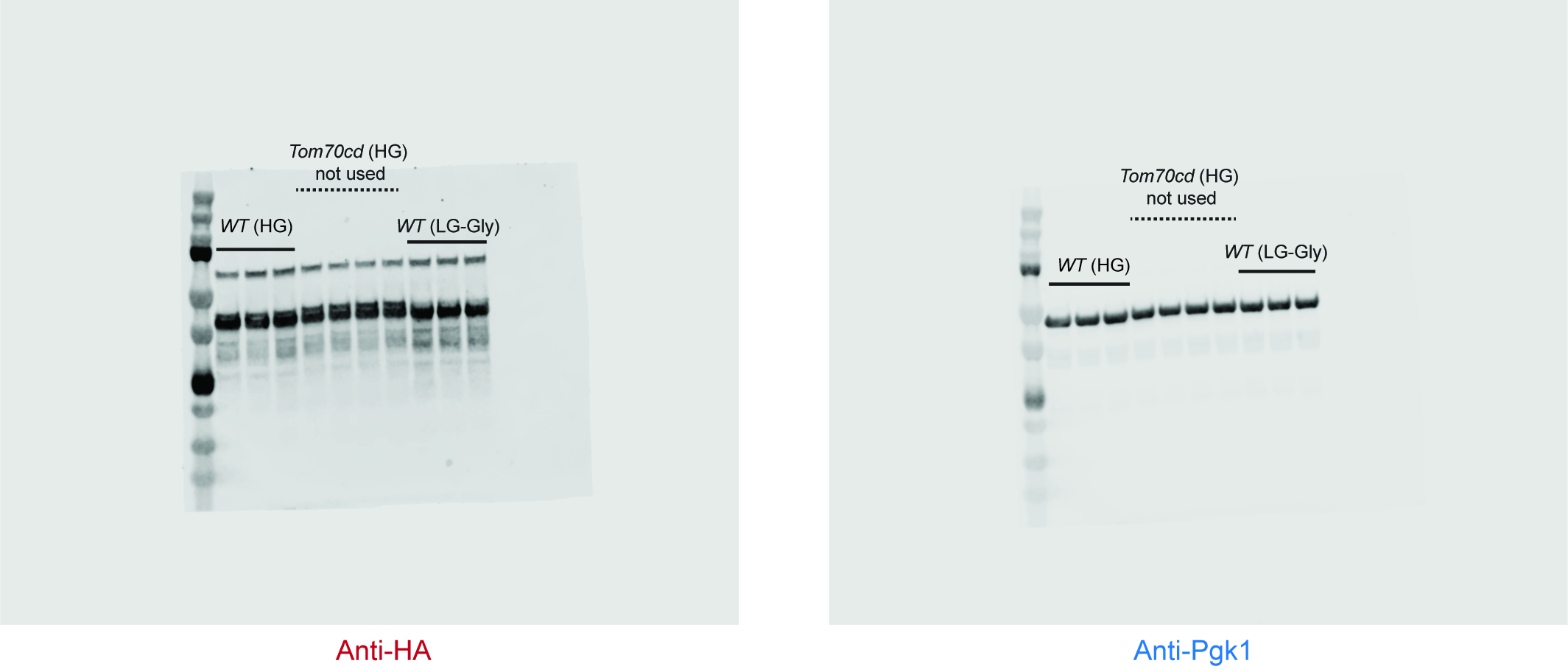

Supplement: Figure 1—figure supplement 2—source data 2. [file elife-87518-fig1-figsupp2-data2.zip › Figure 1-Figure Supplement 2-Source Data 2/WT in HG vs LG-Gly media/Labeled blots.tif]

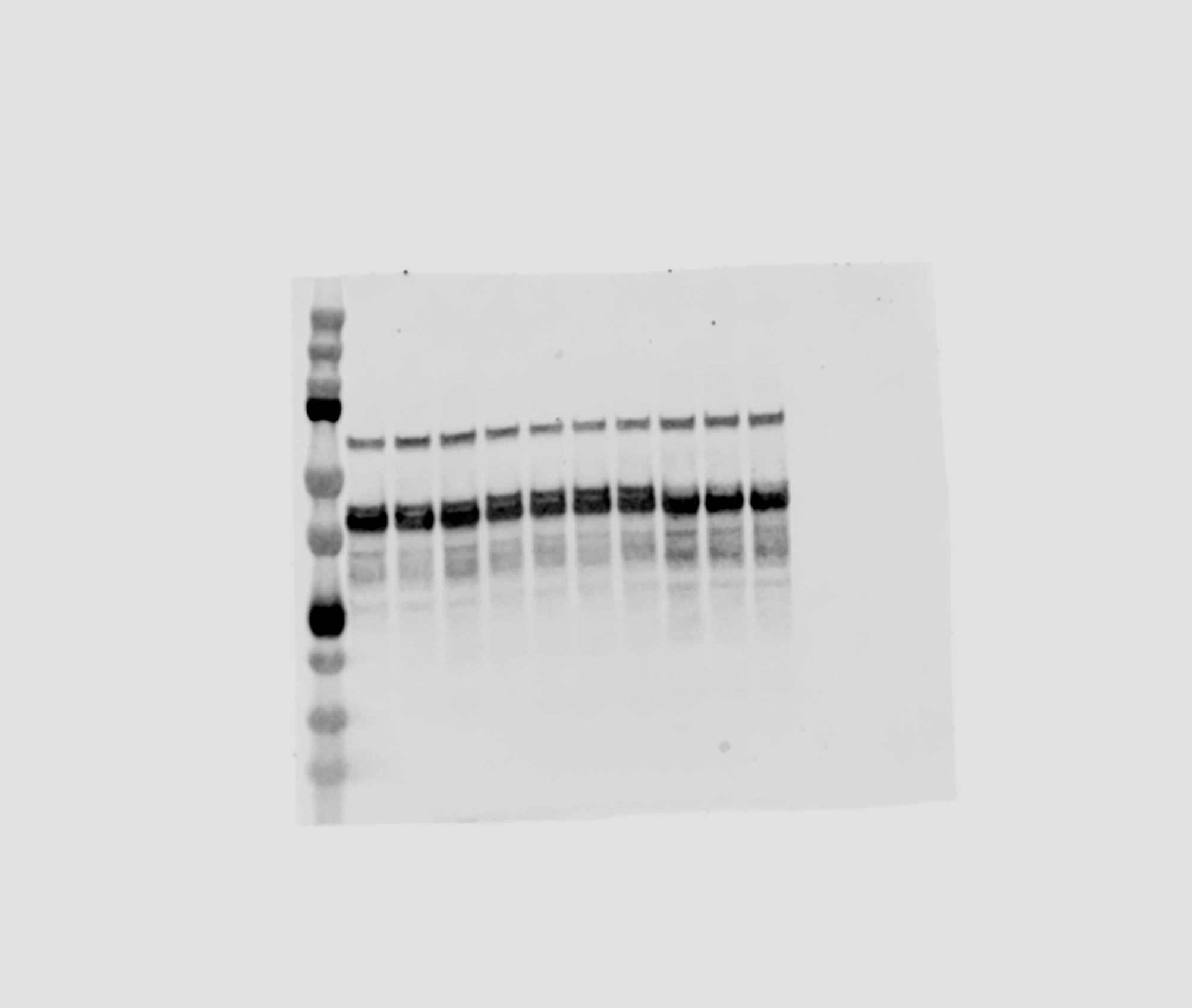

Supplement: Figure 1—figure supplement 2—source data 2. [file elife-87518-fig1-figsupp2-data2.zip › Figure 1-Figure Supplement 2-Source Data 2/WT in HG vs LG-Gly media/WT HG vs WT LG-Gly_anti-HA.tif]

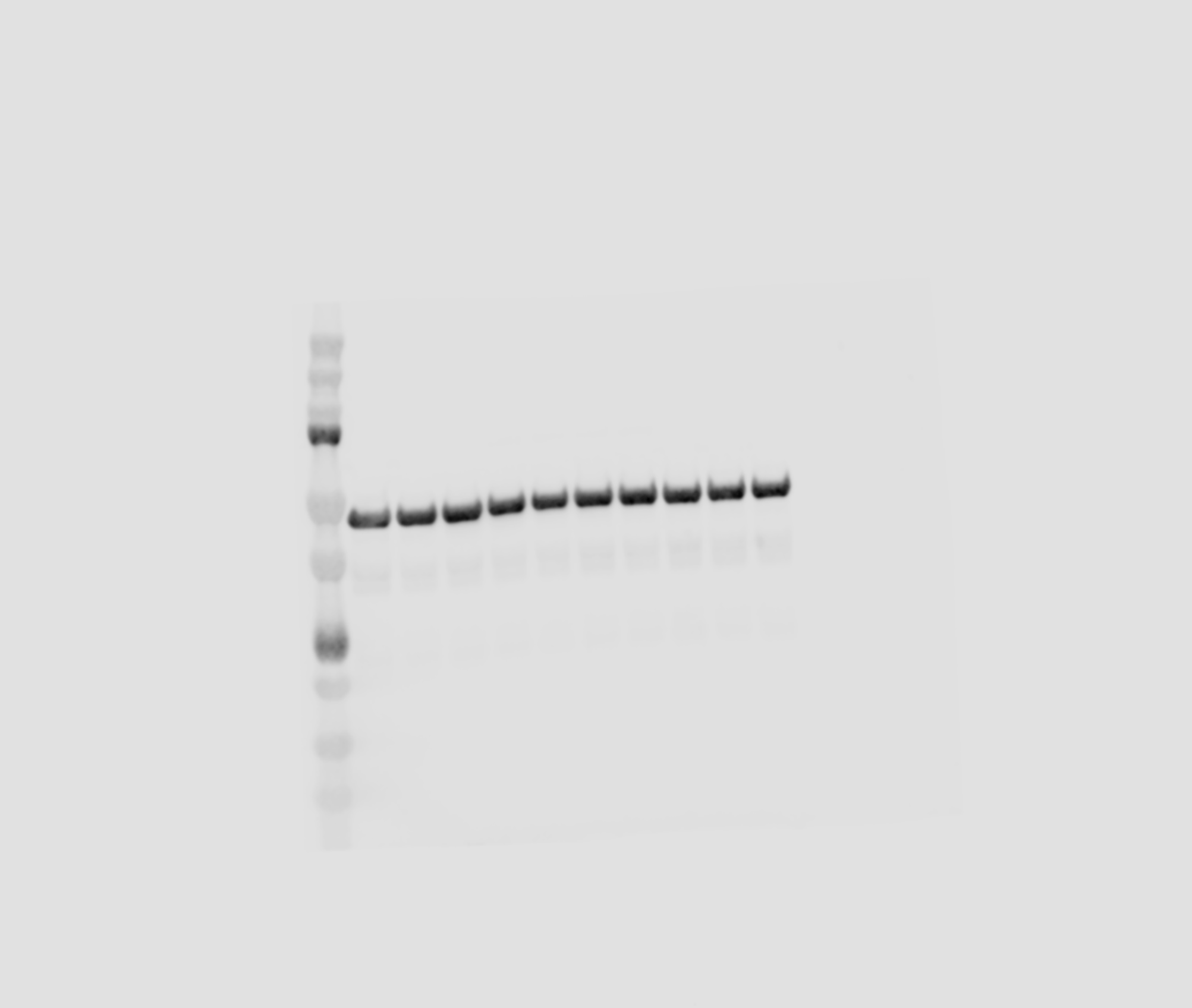

Supplement: Figure 1—figure supplement 2—source data 2. [file elife-87518-fig1-figsupp2-data2.zip › Figure 1-Figure Supplement 2-Source Data 2/WT in HG vs LG-Gly media/WT HG vs WT LG-Gly_anti-Pgk1.tif]

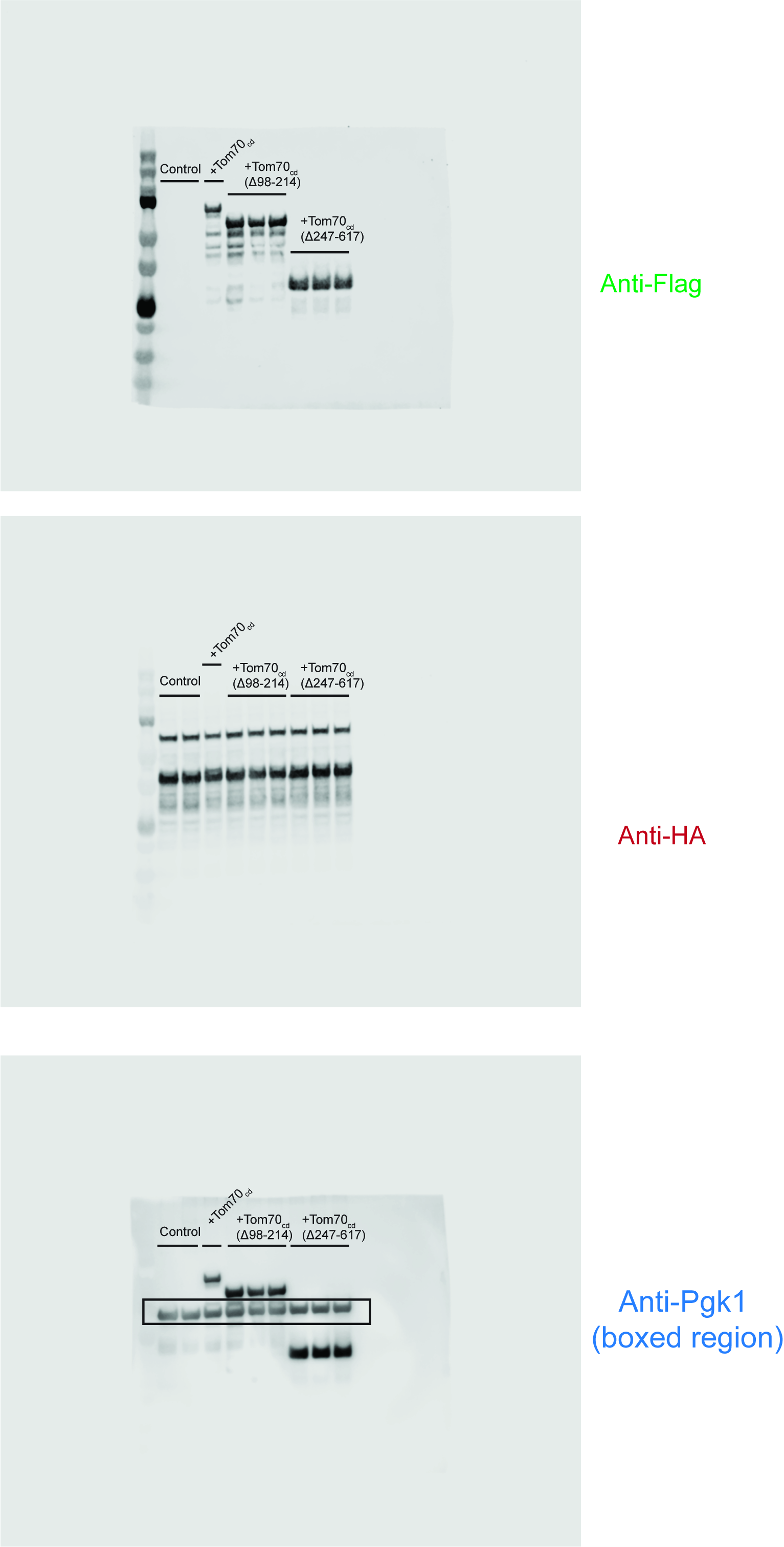

Supplement: Figure 3—figure supplement 1—source data 2. [file elife-87518-fig3-figsupp1-data2.zip › Figure 3-Figure Supplement 1-Source Data 2/Labeled blots.tif]

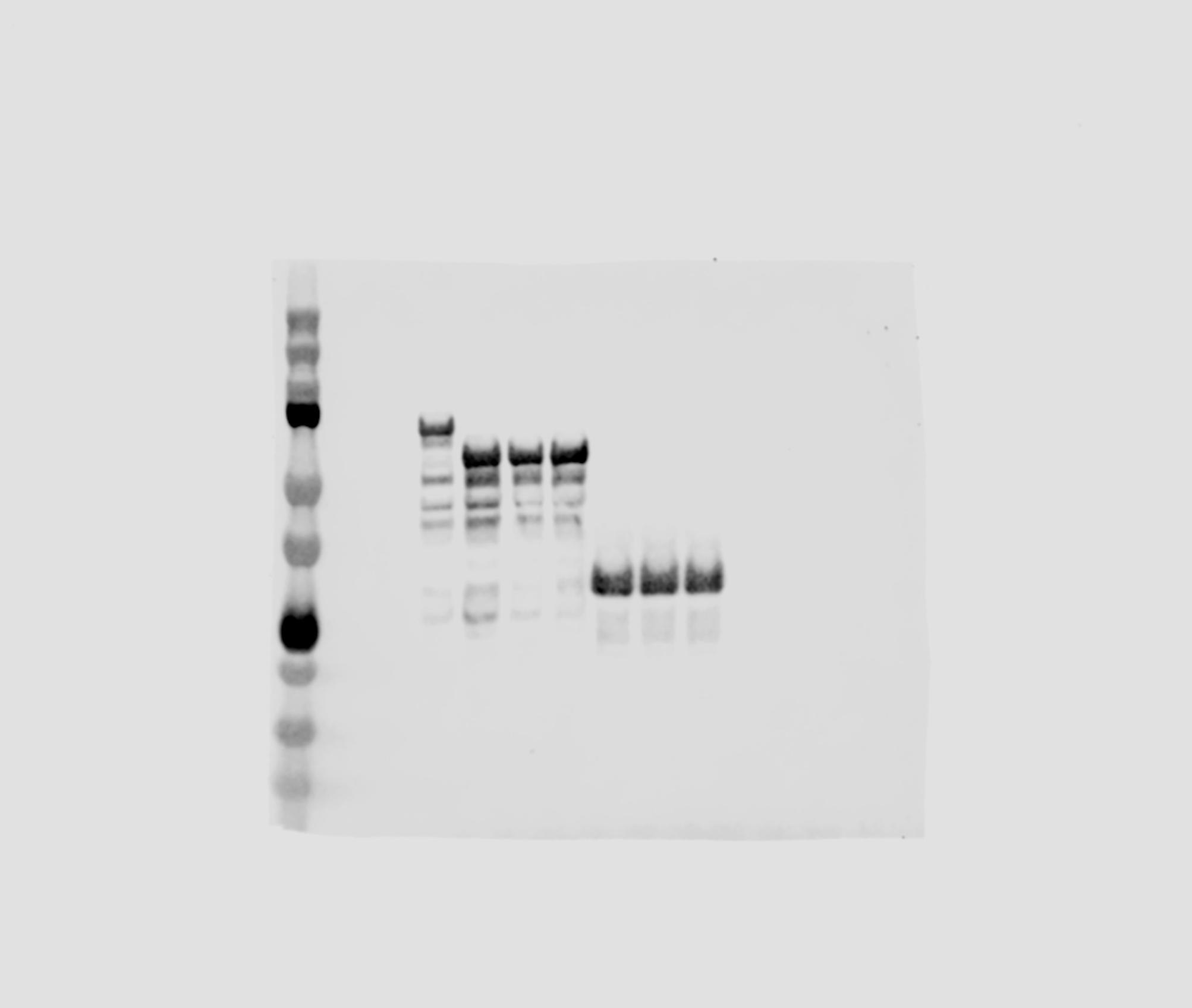

Supplement: Figure 3—figure supplement 1—source data 2. [file elife-87518-fig3-figsupp1-data2.zip › Figure 3-Figure Supplement 1-Source Data 2/anti-Flag.tif]

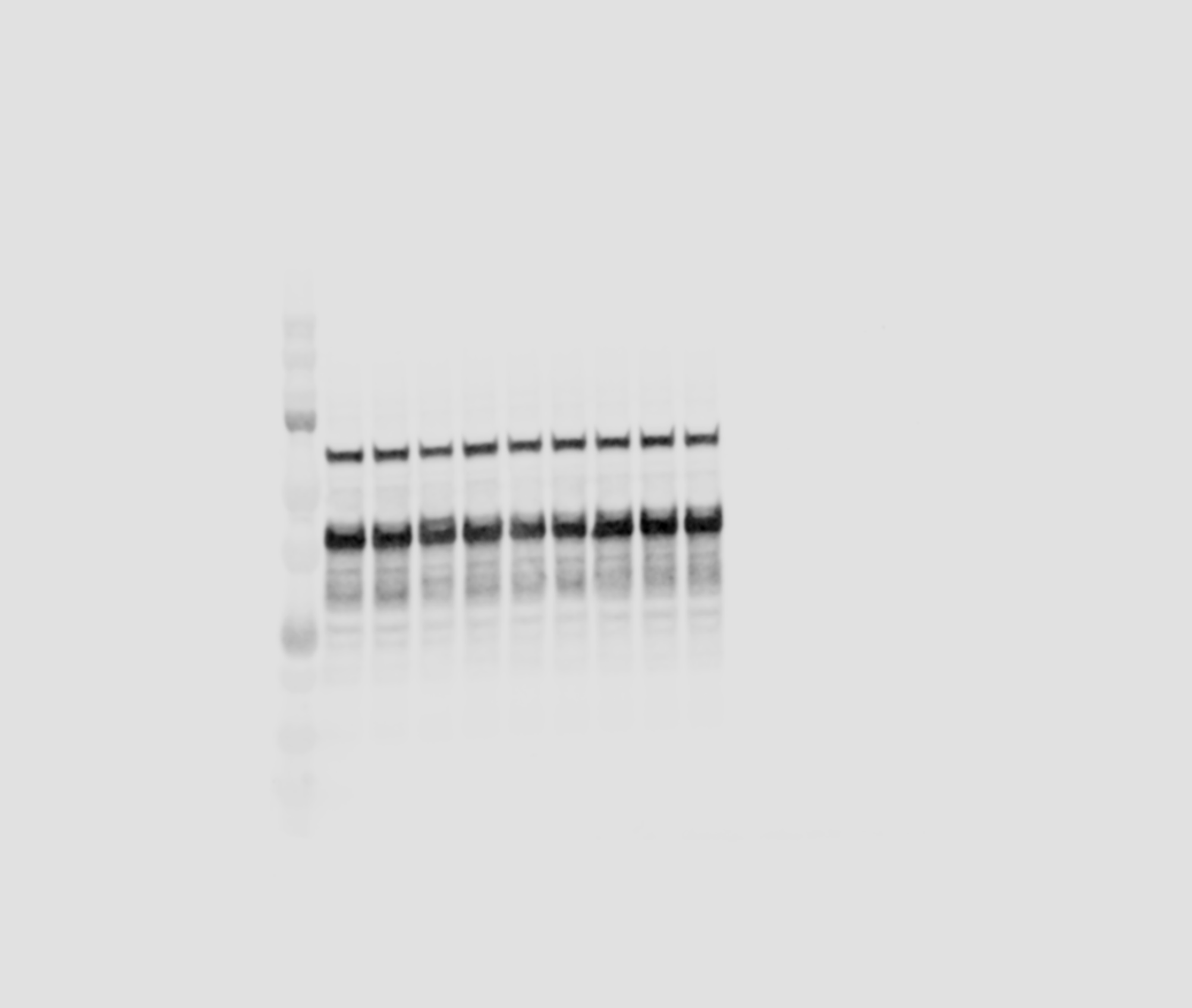

Supplement: Figure 3—figure supplement 1—source data 2. [file elife-87518-fig3-figsupp1-data2.zip › Figure 3-Figure Supplement 1-Source Data 2/anti-HA.tif]

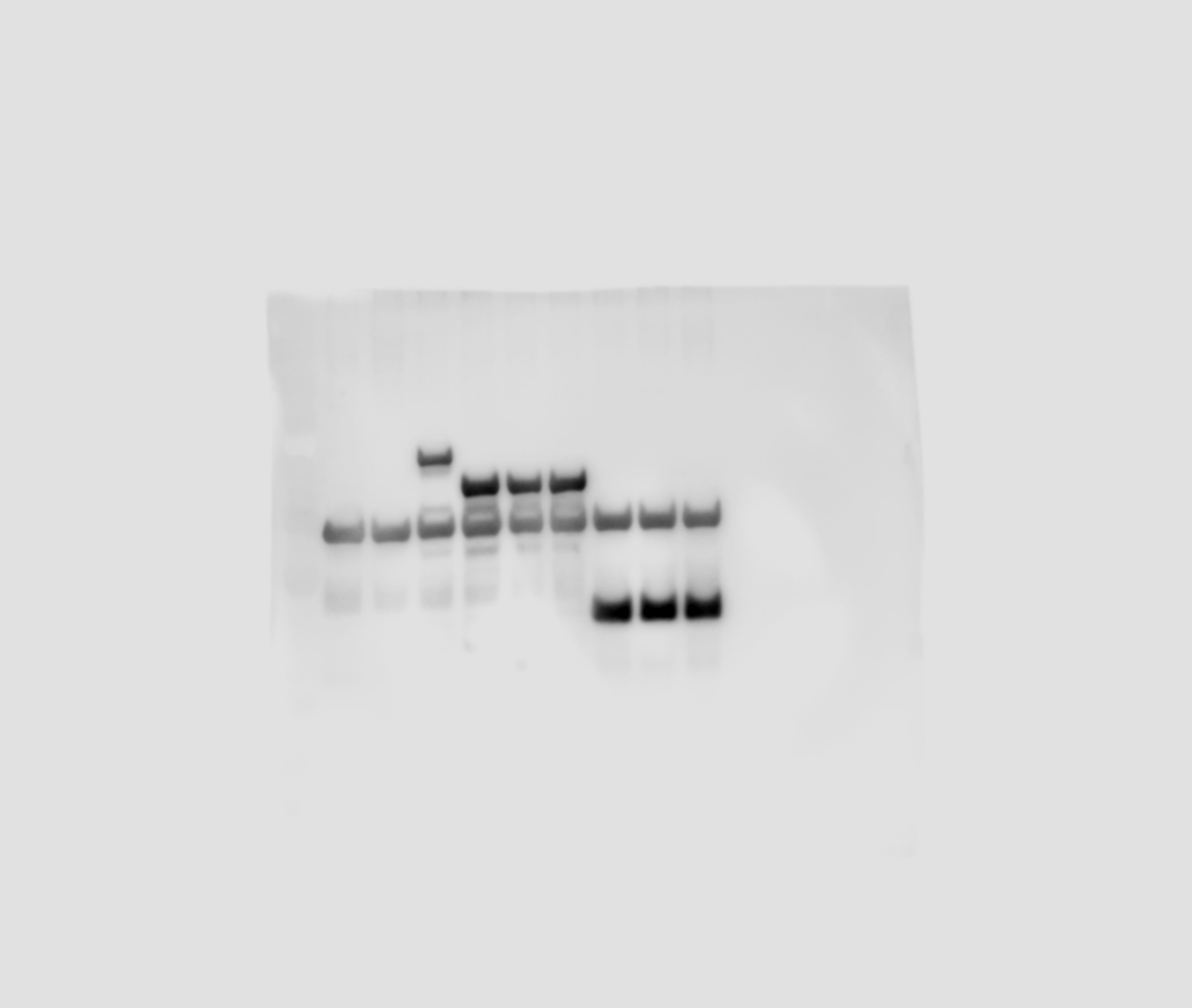

Supplement: Figure 3—figure supplement 1—source data 2. [file elife-87518-fig3-figsupp1-data2.zip › Figure 3-Figure Supplement 1-Source Data 2/anti-Pgk1.tif]
